# Tailoring Fumaric Acid Delivery: The Role of Surfactant-Enhanced Solid Lipid Microparticles via Spray-Congealing

**DOI:** 10.3390/foods13193195

**Published:** 2024-10-08

**Authors:** Yen-Chiu Tsai, Ling Chen, Maoshen Chen, Yun Ma, Fang Zhong, Fei Liu

**Affiliations:** 1 State Key Laboratory of Food Science and Resources, Jiangnan University, Wuxi 214122, China; ayddi.tsai@gmail.com (Y.-C.T.); lingchen@jiangnan.edu.cn (L.C.); chenmaoshen@jiangnan.edu.cn (M.C.); yunma@jiangnan.edu.cn (Y.M.); fzhong@jiangnan.edu.cn (F.Z.); 2 Science Center for Future Foods, Jiangnan University, Wuxi 214122, China; 3 School of Food Science and Technology, Jiangnan University, Wuxi 214122, China; 4 International Joint Laboratory on Food Safety, Jiangnan University, Wuxi 214122, China; 5 Jiaxing Institute of Future Food, Jiaxing 314050, China

**Keywords:** fumaric acid, solid lipid microparticles, spray-congealing, release modulation

## Abstract

Fumaric acid, a naturally occurring preservative with antimicrobial properties, has been widely used in the baking industry. However, its direct addition interferes with yeast activity and negatively impacts the gluten structure. This study investigates the potential of spray-congealing as a method for encapsulating fumaric acid within solid lipid microparticles. The selection of lipid carriers and surfactants is critical, so hydrogenated palm stearin, hydrogenated rapeseed oil, and Compritol ATO 888 (glyceryl behenate) were chosen as lipid carriers, and propylene glycol monostearate and glyceryl monolaurate were utilised as surfactants with varying concentrations. Rheological properties, encapsulation efficiency, particle size, moisture content, and thermal behaviour were assessed, along with the release profiles under different temperature conditions simulating the baking process. The findings indicate that the addition of surfactants significantly impacts the viscosity and stability of the molten mixtures, which in turn affects the spray-congealing process and the release of fumaric acid. The temperature-dependent and time-dependent release profiles demonstrate the potential for customising release kinetics to suit specific applications, such as the baking industry. This study may contribute to the development of a controlled-release system that synchronises with the baking process, thereby optimising fumaric acid’s functionality while preserving the quality of baked goods.

## 1. Introduction

In the realm of food science, microencapsulation has become a cornerstone for enhancing the functionality of bioactive compounds within food products. The technique offers a means to protect sensitive ingredients, control their release, and ensure their stability throughout the shelf life of food items. One such application is in the baking industry, where microencapsulation can be leveraged to deliver ingredients at specific stages of the baking process, thereby optimising their functionality [1].

Fumaric acid, a naturally occurring organic acid with a distinctive molecular structure consisting of two carboxyl groups (−COOH) attached to a butane chain, has been widely recognised for its preservative properties. Its ability to inhibit mould growth makes it an attractive candidate for enhancing the shelf life of food products [2]. The antimicrobial activity of fumaric acid is attributed to its ability to lower the pH of the environment, disrupt cell membrane integrity, and interfere with essential metabolic pathways in microorganisms [3]. Acidified bread, which incorporates fumaric acid as a means of natural preservation, has been shown to extend the shelf life of baked goods. By creating an acidic environment, fumaric acid inhibits the growth of spoilage microorganisms, thus maintaining the quality and safety of bread over an extended period [4,5]. The use of fumaric acid in bread not only serves as a natural preservative but also contributes to the bread’s texture and flavour profile, making it a desirable ingredient in various types of baked products [6].

The encapsulation of fumaric acid is particularly pertinent in this context. While it offers significant benefits, its early release during the baking process can interfere with yeast activity and negatively impact the gluten structure, affecting the texture and quality of the final product [7]. To mitigate these drawbacks, the controlled release of fumaric acid in the later stages of baking is essential. Spray-congealing and using hot melt particles, or fluid-bed coating, are the leading encapsulation processes in the baking industry due to their customisable release profiles and ability to protect ingredients. While fluidised-bed coating creates a reservoir-like microcapsule, it may lead to an uncontrolled burst release if the shell’s integrity is compromised [8,9]. Spray-congealing, on the other hand, disperses the active ingredient within a matrix, offering a more controlled release and minimizing the risk of premature release during storage and handling. This method is particularly beneficial for fumaric acid encapsulation, ensuring its release aligns with the baking process, thus preserving yeast activity and maintaining the gluten structure [7,10]. Despite the availability of other techniques such as extrusion embedding, spray-drying, and liposome entrapment, they are less common due to their specific demands and complexities to meet the functional and quality requirements of bakery products [10,11].

The present study aims to explore the potential of spray-congealing as a method for microencapsulating fumaric acid, focusing on the development of a controlled-release system that aligns with the baking process. By encapsulating fumaric acid within solid lipid microparticles (SLMs), the release of the acid can be delayed until the later stages of baking, thus minimising its interaction with yeast and preserving the desirable gluten structure. The choice of lipid carriers and surfactants is critical, as they influence the encapsulation efficiency, particle size distribution, and the release profile of the encapsulated acid. A number of studies investigated the effect of surfactants in drug release modulation through microencapsulation, though they were mostly focused on spray-drying [12,13,14]. Specifically, John & Becker [15] found that surfactant, specifically sorbitan monooleate, had a concentration-dependent effect on the dissolution rate. It could be used to modulate drug release, with higher concentrations potentially causing a delay in drug release in acidic conditions and promoting release in alkaline conditions. Ouyang et al. [16] concluded that surfactants played a significant role in the spray-congealing process by reducing the viscosity of the molten wax, improving drug embedment, and modulating drug release. The use of surfactants enabled the successful embedment of hydrophilic paracetamol within spray-congealed microparticles, which is beneficial for taste-masking and sustained drug release application [17,18,19].

In this context, hydrogenated palm stearin (HPO), hydrogenated rapeseed oil (HRO), and Compritol ATO 888 (COM) are lipid carriers chosen for their distinct chemical structures and physicochemical properties, making them ideal for use in solid nanoparticles aimed at controlled release applications. HPO, derived from the hydrogenation of palm oil’s stearin fraction, is characterized by a high content of saturated fatty acids, predominantly palmitic and stearic acids, which confer a higher melting point and stability [20,21]. HRO results from the hydrogenation of rapeseed oil, a source naturally rich in oleic and linoleic acids, leading to a solid fat with a reduced level of unsaturation and enhanced stability [22,23]. Compritol ATO 888, a blend of behenic acid esters, is known for its high melting point and stability, attributed to the fully saturated behenic acid within its structure [24,25,26]. These lipids form the backbone of solid nanoparticles, providing a controlled release matrix for various applications while ensuring the stability and physical characteristics necessary for effective delivery systems. Additionally, propylene glycol monostearate (PGM) and glyceryl monolaurate (GML) are utilised as surfactants to modulate the viscosity and stability of the molten mixtures, which in turn affect the spray-congealing process and release profile. The experimental design involves the preparation of molten mixtures with varying concentrations of PGM and GML, followed by spray-congealing to form fumaric acid microparticles (FAMP). The rheological properties of these mixtures, including their viscosity and temperature dependence, are characterised to understand their behaviour during the spray-congealing process. The encapsulation efficiency, particle size, and moisture content of the resulting microparticles are also assessed to evaluate their potential for the controlled release of fumaric acid. Furthermore, the thermal behaviour of the microparticles, as analysed by differential scanning calorimetry (DSC), and their release profiles under different temperature conditions simulate the baking process. This allows for the evaluation of the microparticles’ performance in terms of the controlled release of fumaric acid, ensuring that the acid is released at the optimal time to preserve the bread’s quality without compromising its texture. The findings of this study are expected to contribute to the development of innovative food products as well as more efficient utilisation of the role of microencapsulation in controlled release applications within the food industry.

## 2. Materials and Methods

### 2.1. Materials

Hydrogenated rapeseed oil (HRO) was kindly provided by Yihai Kerry Co., Ltd., Shanghai, China. Hydrogenated palm stearin (HPO) and Compritol 888 ATO (COM) were obtained from a local chemical distributor at Tekang Biotechnology Co., Ltd., Henan, China, and Gattefossé, Shanghai, China. Fumaric acid was manufactured and provided by ZiBoJuSiTe Biotechnology Co., Ltd., Shangdong, China. Food-grade gyceryl monolaurate (GML) and propylene glycol monostearate (PGM) were manufactured and provided by Guangzhou Kevin Food Development Co., Ltd, Guangdong, China. All chemicals and reagents were of analytical grade.

### 2.2. Preparation of Molten Mixture for Rheological Tests

A total of 27 molten mixture formulations were prepared for rheological examination and subsequently spray-congealed into FAMP. The three lipid carriers selected were HPO, HRO, or COM, with each FAMP comprising one lipid carrier, fumaric acid, and varying proportions of PGM/GML. All formulations were designed to encapsulate fumaric acid at a level of 50% *w*/*w*, calculated based on the total weight of the encapsulated drug and the coating materials combined. The surfactants were incorporated at concentrations of 5%, 10%, 15%, and 20% *w*/*w* of the entire formulation. These specific concentrations were determined following preliminary experiments, with the objective of tailoring the microparticles for application in bread production.

Briefly, the required amounts of each material were accurately weighed according to the formulation into a beaker and then transferred into a water bath maintained at a temperature above the melting point (Appendix A, Table A1) of the respective lipid carrier (HPO, HRO, or COM) for 15 min and stirred to form homogeneous molten mixtures.

### 2.3. Rheological Tests of Molten Mixture

#### 2.3.1. Continuous Ramping Tests

The methods were adapted from Wong et al. [27]. In brief, a rheometer (AR-G2, TA Instruments, New Castle, DE, USA) with a parallel plate system (20 mm diameter, gap 200 μm) was used to determine the viscosity of the molten formulations at different shear stresses. Briefly, samples were heated to a temperature of 10 °C above their respective peak melting temperatures (Appendix A, Table A1) and held at that particular temperature for an equilibration time of 5 min. The samples were then sheared at an increasing shear stress from 10 to 100 Pa over a time duration of 5 min to obtain the rheograms.

#### 2.3.2. Temperature Ramping Tests

A rheometer (AR-G2, TA Instruments, New Castle, DE, USA) was used to investigate the viscosity–temperature relationship. Samples were heated from their respective peak melting temperatures (Appendix A, Table A1) to 100 °C at a constant shear stress of 5 Pa over a time duration of 5 min. Viscosity values were recorded at different temperatures.

### 2.4. Production of FAMP

A total of 27 formulations were spray-congealed into FAMP. The three lipid carriers selected were HPO, HRO, or COM, with each FAMP composed of one lipid carrier and fumaric acid alongside varying proportions of PGM/GML. All formulations were designed to encapsulate fumaric acid at a level of 50% *w*/*w* based on the total weight of the encapsulated drug and the coating materials combined. The surfactants were incorporated at concentrations of 5%, 10%, 15%, and 20% *w*/*w* of the entire formulation. These specific concentrations were chosen following preliminary experiments, with the aim of tailoring the microparticles for application in bread.

A laboratory-scale spray congealer (two-fluid nozzle, B-290, Buchi, Switzerland) was set up. A two-fluid nozzle, stainless steel, with a 0.7 mm nozzle tip was employed for the atomisation of the molten material. An atomising pressure of 0.2 bar was used with the cooling chamber maintained at an ambient room temperature of around 20 °C. Atomisation air temperature was maintained above the melting point of the lipid carrier. The molten material and circulation water bath were maintained above the melting temperature of the lipid material. The molten mixtures were conveyed to the spray nozzle via a peristaltic pump at a rate of 37 mL/min. The microparticles obtained from the spray-congealing process were passed through a 100-mesh sieve and subsequently stored in an airtight plastic container within a desiccator for subsequent analysis.

### 2.5. Characterisation of FAMP

#### 2.5.1. Determination of Total Acid Content, Encapsulation Efficiency (EE), and Loading Capacity

The quantification of the released acid was conducted using a standardised titration method with reference to the Chinese Standard of GB 25546 [28]. For the determination of total fumaric acid content, 0.4 g of the FAMP was measured and placed into a 250 mL conical flask. The FAMP was completely dissolved using 20 mL of chloroform, followed by dilution with 10 mL of 75% ethanol. Phenolphthalein was added as an indicator, in two to three drops, and the solution was titrated with a 0.5 mol/L sodium hydroxide (NaOH) solution. The endpoint of titration was identified when a faint red colour persisted for 15 s without change. The volume of NaOH solution used, denoted as *V*_1_, and a blank titration volume, *V*_0_, were both recorded.

(1) $$Acid\;Content\;\left(\%\right)=\frac{\frac{\left(V_{1}-V_{0}\right)}{1000}\times C\left(NaOH\right)\times 58.04}{0.4}\times 100,$$

where *V*_1_ is the volume of NaOH solution required for all the fumaric acid present in the sample, *V*_0_ is the initial blank titration volume, C(NaOH) is the concentration of the NaOH solution, and 58.04 is the molar mass of fumaric acid.

For the encapsulation efficiency assessment, another 0.4 g of FAMP was weighed and placed into a 100 mL beaker. The mixture was dissolved in 20 mL of 75% ethanol with 30 s of shaking. Afterward, the mixture was filtered through a quantitative filter paper (2.5 µm, 15 cm diameter, medium speed, Sinopharm, Shanghai, China) into a 250 mL conical flask. The beaker was rinsed with three portions of 10 mL of 75% ethanol, with each rinsing followed by shaking and filtering. The filtrates were combined in the conical flask, and the solution was treated with two to three drops of phenolphthalein, then titrated to a faint red colour that remained unchanged for 30 s using the standardised 0.5 mol/L NaOH solution. The volume of NaOH solution used in this titration, *V*_2_, and a new blank titration volume, *V*_0_′, were recorded.

(2) $$Encapsulation\;Efficiency\;\left(\%\right)=\frac{\left(V_{1}-V_{0}\right)-\left(V_{2}-V_{0}\prime \right)}{\left(V_{1}-V_{0}\right)}\times 100,$$

where *V*_1_ is the volume of NaOH solution used to determine the total acid content, *V*_0_ is the blank titration volume, *V*_2_ is the volume of NaOH solution used to determine the free acid content not encapsulated within the microparticles, and *V*_0_′ is the blank titration volume.

The loading capacity, which indicates the percentage of fumaric acid that is effectively encapsulated within the microparticles, is derived from the difference between the total acid content (*V*_1_) and the free acid content (*V*_2_).

#### 2.5.2. Particle Size Analysis

The particle size distribution was analysed by Laser Diffraction using a Malvern Mastersizer 3000 equipped with a wet dispersion unit (Malvern, UK). Samples were analysed averaging five replicates at 25 °C using distilled water as the dispersant medium. The size span, *Sx*, was calculated as follows:

(3) $$Sx=\frac{d_{90}-d_{10}}{d_{50}},$$

where *d*_10_, *d*_50_, and *d*_90_ are the diameters at the 10th, 50th, and 90th percentiles of the cumulative particle size distribution, respectively. The size span categories are the spread of particle size distributions, where a higher value represents a broader size distribution.

#### 2.5.3. Hot Stage Microscopy

Fumaric acid-loaded (50%, *w*/*w*) FAMP samples were examined using a hot-stage microscope (BX51, Olympus Optical, Tokyo Japan) with a heating unit. A small amount of FAMP was scattered on a glass slide and heated at a rate of 5 °C/min, with the starting temperature set at 35 °C, which marks the average highest proofing temperature in the baking industry. Changes in the FAMP with temperature were monitored by capturing timed images that detailed the entire melting process.

### 2.6. Differential Scanning Calorimetry (DSC)

The thermal characteristics of FAMP were determined using a differential scanning calorimeter (TA Instruments, Alzenau, Germany) with an empty pan as a reference. A hermetically sealed aluminium pan loaded with approximately 5 mg of the sample was placed in a DSC furnace. Analyses were conducted one day after FAMP production, and scans were performed between 25 °C and 200 °C at a rate of 5 °C/min in an inner atmosphere of nitrogen gas at a flow rate of 25 mL/min after equilibration at 25 °C for 5 min. Uncoated fumaric acid and lipid materials were also analysed for comparison.

### 2.7. Powder X-ray Diffraction (XRD) Analysis

X-ray powder diffractometers elucidated the polymorphic profiles of the unprocessed materials, physical mixtures, and spray-congealed SLMs with Cu Kα radiation (λ = 1.5406 Å). The crystallinity of the constituents (lipid carriers, surfactants, and fumaric acid) and microparticles were determined by XRD in Bruker D2 Phaser equipment. The voltage and current were 30 kV and 10 mA, respectively. The data collection was made in 2θ step scan mode with a scanning rate of 2° (2θ)/min in the angle ranged from 10 to 50°.

### 2.8. Flourier Transform-Infrared Spectroscopy

Interactions between Fumaric acid and lipids as well as additives were investigated using Fourier transform-infrared (FTIR) spectroscopy. The attenuated total reflection (ATR) method was utilized. The prism surface was first cleaned with 90% ethanol and dried using lint-free tissue. A background reading was first taken, followed by samples of approximately 20 mg, placed on the clean prism surface and compressed. Infrared spectra of the samples were obtained and analysed. The prism surface was cleaned using 90% ethanol in between samples. Interaction among the lipid carriers and FA in the microparticles was investigated using FT-IR spectroscopy (Spectrum 100, Perkin Elmer, CT, USA).

### 2.9. Release Studies

#### 2.9.1. Temperature-Sensitivity

An in vitro approach was employed to characterise the release profiles of FAMP under controlled temperature conditions, simulating the baking process. The evaluation specifically focused on how varying temperatures affect the release dynamics of the encapsulated fumaric acid. The temperatures selected for this study, 35 °C, 53 °C, 67 °C, and 90 °C, represent critical stages in the baking cycle from the final proofing to the completion of baking. The methodology is designed to provide a clear and systematic framework for assessing the release behaviour of FAMP under conditions that mimic the baking process.

A total of 0.018 g of FAMP was weighed into a 15 mL centrifuge tube. Deionised water, serving as the dispersing medium analogous to the baking dough’s aqueous environment, was added to achieve a final volume of 15 mL. The mixture was then vortexed for 20 s to ensure complete dispersion of the microparticles. Following this, the samples were incubated in a thermostatic water bath heater (Yuejin Medical Apparatus, Shanghai, China) for 15 min at specific temperatures designed to reflect various stages of baking. Post-incubation, the samples underwent filtration through a filter paper (2.5 µm, 15 cm diameter, medium speed, Sinopharm, Shanghai, China) to isolate the released fumaric acid. The quantification of the released acid was conducted as in Section 2.5.1.

#### 2.9.2. Time-Dependent Release

A total of 0.018 g of FAMP was weighed into 15 mL centrifuge tubes, 15 mL of 20 °C distilled water was added to each tube, and the mixture was vortexed for 20 s to ensure uniform dispersion. The samples were then incubated in a hot water bath at either 35 °C or 90 °C—temperatures that correspond to the proofing and baking stages of bread production. Samples were collected at specific intervals—5, 10, 30, 60, and 90 min at 35 °C, and 1, 3, 6, 9, 12, and 15 min at 90 °C—to mimic the proofing intervals and baking times at each temperature, respectively. Each collected sample was filtered through a filter paper (2.5 µm, 15 cm diameter, medium speed, Sinopharm, Shanghai, China), and the released fumaric acid was quantified using the titration method identical to Section 2.5.1.

### 2.10. Statistical Analysis

An ANOVA at a 5% level of significance was conducted using PRISM software v.10 to assess the differences among group means where appropriate. Each mixture was prepared and analysed in triplicate, with a minimum of three independent repetitions of the mixture preparation process. The data were considered statistically significant if *p* < 0.05. The ANOVA was specifically used to test for statistically significant differences between the mean values of the groups compared in this study.

## 3. Results

### 3.1. Rheological Tests of Molten Dispersion

#### 3.1.1. Flow Ramping Test

The flow ramping test data for molten feeds in spray-congealing processes were analysed to investigate the rheological behaviour of mixtures containing 50% fumaric acid and three different lipid carriers: hydrogenated palm stearin (HPO), hydrogenated rapeseed oil (HRO), and Compritol (COM). The addition of surfactants PGM and GML was also examined to understand its influence on the systems’ rheology.

**Figure 1:**
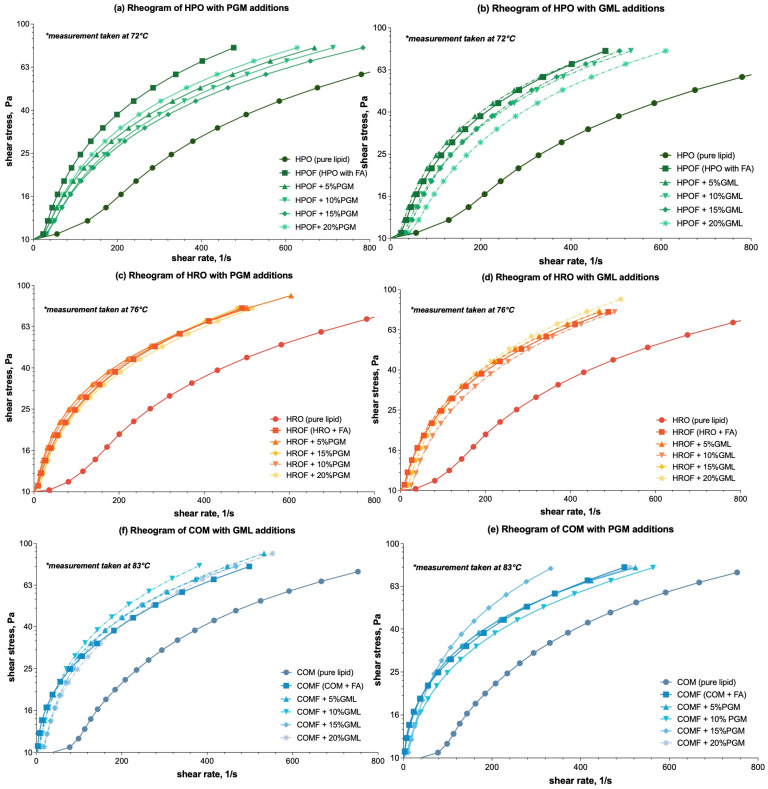
Rheograms of molten mixtures of HPO-based (Hydrogenated Palm Stearin), HRO-based (Hydrogenated Rapeseed Oil), and COM-based (Compritol ATO 888) lipid carriers with additions of PGM/GML in the formulations: (**a**) Effect of PGM on HPO-based formulations; (**b**) Effect of GML on HPO-based formulations; (**c**) Effect of PGM on HRO-based formulation; (**d**) Effect of PGM on HRO-based formulations; (**e**) Effect of PGM on COM-based formulations; (**f**) Effect of GML on COM-based formulations. For ease of differentiation, the three lipid and derivative formulations are color-coded: HPO-based formulations are represented by green lines, HRO-based formulations by orange/red lines, and COM-based formulations by blue lines. GML formulations were further distinguished with dashed lines to contrast with the solid lines used for PGM formulations. * Temperature of the rheology test that corresponded to 10 °C above the melting point (Appendix A, Table A1) of the lipid carrier was set.

The rheograms for HPOF, HROF, and COMF demonstrated a consistent increase in shear stress with constant shear rate, indicative of pseudo-plastic or shear-thinning behaviour [29], as demonstrated in the following: Figure 1a,b—HPO (hydrogenated palm stearin) → HPOF (fumaric acid spray-congealed with HPO); Figure 1c,d—HRO (hydrogenated rapeseed oil) → HROF (fumaric acid spray-congealed with HRO); and Figure 1e,f—COM → COMF (fumaric acid spray-congealed with COM). This behaviour is characterized by a decrease in apparent viscosity with increasing shear rate, a common trait in complex fluids like polymer solutions and melts.

The addition of PGM and GML to HPOF was observed to increase viscosity, as evidenced by higher shear stress values at equivalent shear rates. For example, with reference to Figure 1a, at a shear stress of 10.60 Pa, the inclusion of 5% PGM in HPOF increased the viscosity value from the original viscosity to 24.84 Pa. This represented a substantial increase in resistance to flow, suggesting a potential yet to be proven correlation between surfactant concentration and viscosity.

For HROF, the incorporation of PGM and GML also resulted in deviations from HROF’s viscosity, as evidently shown in Figure 1c,d. However, the specific data points from the trials were not significant enough, i.e., *p* ≥ 0.05, to warrant a detailed comparison. The observed increases in shear stress values with the addition of surfactants suggest a trend consistent with HPO, where viscosity augments with surfactant concentration.

The effects of PGM and GML on COMF were particularly pronounced, with a marked changes in viscosity as illustrated in Figure 1e,f, especially with GML. At a shear stress of 10.73150 Pa, the incorporation of 5% GML into COMF resulted in a significant rise in viscosity. Initially presenting a viscosity value of 10.27 Pa, the addition of GML elevated this value to 20.36 Pa. This enhancement reflects an increase of 10.09 Pa, underscoring the substantial impact of GML on COMF’s viscosity. This comparison vividly illustrates the pronounced effect of GML on COMF’s rheological behaviour, suggesting a shift from the initial viscosity to a higher, more resistant state.

When comparing the effects of PGM and GML across the lipid carriers, it became clear that the influence of these surfactants on viscosity was not consistent. For COMF, GML had a notably more substantial impact on viscosity than PGM, as evidenced by the viscosity values at equivalent concentrations. In contrast, for HROF, the addition of surfactants did not significantly alter viscosity (*p* ≥ 0.05), suggesting that other factors, such as the presence of fumaric acid, may be more influential in this system and warrant further investigation.

In summary, the effects of surfactants PGM and GML on viscosity were dependent on the lipid carrier type. While both surfactants had noticeable effects on HPOF and COMF, their impact on HROF was less pronounced. The rheograms provided a clear visual representation of these trends, with distinct variations in shear stress and shear rate that suggest potential non-Newtonian behaviour. Notably, the presence of fumaric acid had a distinct effect on viscosity across all lipid carriers, including HROF, where it appeared to be the primary driver of viscosity changes rather than the surfactants. These emphasize the importance of considering the specific interactions between surfactants, lipid carriers, and the encapsulated material, such as fumaric acid, when predicting rheological properties.

#### 3.1.2. Temperature Ramping Test

**Figure 2:**
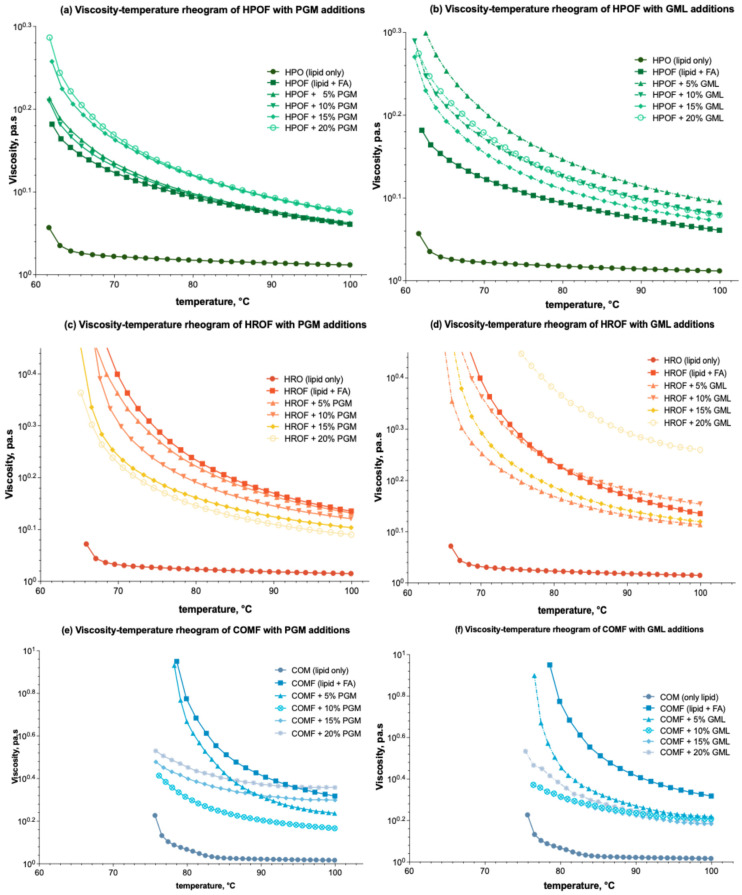
Viscosity-temperature rheograms of HPO-based, HRO-based, and COM-based lipid carrier with additions of PGM/GML in the formulations: (**a**) Effect of PGM on HPO-based formulation; (**b**) Effect of GML on HPO-based formulation; (**c**) Effect of PGM on HRO-based formulation; (**d**) Effect of GML on HRO-based formulation; (**e**) Effect of PGM on COM-based formulation; (**f**) Effect of GML on COM-based formulation.

The temperature-dependent viscosity of molten feeds in spray-congealing is critical for controlling droplet size and microparticle formation. The integration of fumaric acid into lipid carriers exerts a notable influence on viscosity across a range of temperatures, as depicted in Figure 2a,b for HPO (HPO → HPOF), Figure 2e,f for HRO (HRO → HROF), and Figure 2e,f for COM (COM → COMF).

As shown in Figure 2a,b, the introduction of both PGM and GML additives into HPOF leads to a modest increase in viscosity, yet this effect does not appear to be markedly temperature-dependent.

In the case of HRO, as reflected through Figure 2c, the addition of PGM results in a decrease in viscosity, particularly at lower temperatures (~60 °C). Conversely, GML reduces viscosity at concentrations below 15% but shows an increase at the 20% concentration level, as shown in Figure 2d. This observation contradicts the expectation of a continuous decrease in viscosity with surfactant addition and thus warrants a literature search for plausible explanations. It is hypothesized that at higher concentrations, surfactants may interact differently with the lipid carrier, potentially leading to structural changes that affect viscosity [30].

Turning to COM, or Figure 2e,f, the addition of PGM and GML effectively diminishes the viscosity of the fumaric acid-enriched molten mixtures across all tested concentrations. However, similar to the HRO system, there is a rebound in the viscosity-modifying effect when the surfactant concentration surpasses 15%. It is also worth noting that all formulations display a relatively flat second phase in their temperature-viscosity profiles, indicative of the temperature sensitivity of these systems. For COM, as depicted in Figure 2e,f, the addition of PGM and GML generally reduces viscosity across all tested concentrations. However, a rebound in the viscosity-modifying effect is observed when surfactant concentrations exceed 15%. This behaviour may be attributed to the surfactants’ ability to alter the microstructure of the molten mixtures, which could become more temperature-sensitive at higher concentrations [31].

The literature suggests that surfactants can have complex effects on the viscosity of lipid-based systems, with their influence being dependent on factors such as concentration, temperature, and the specific lipid carrier [32]. The observed increase and decrease in apparent viscosity at lower and higher concentrations of PGM and GML, respectively, may be related to the critical micelle concentration and the resulting changes in the system’s microstructure [33].

### 3.2. Characterisation of Spray-Congealed Microparticles

Given the slightly hydrophobic nature of fumaric acid, this part focuses on exploring ternary formulations with PGM and GML with the aim to (1) enhance the process of spray-congealing and (2) modulate the release of encapsulated acid for applications in baking products.

#### 3.2.1. Acid Content, Efficient Load and Encapsulation Efficiency

The spray-congealing process was utilized to produce microparticles using three distinct lipids and two surfactants at various concentrations, resulting in 29 unique formulations. Notably, as shown in Table 1, all formulations achieved a fumaric acid content of 50% (*w*/*w*) or higher, aligning with the theoretical yield [34,35]. This consistency suggests a robust process that reliably incorporates the active ingredient, irrespective of surfactant presence.

The encapsulation efficiency, which is a measure of the successful encapsulation of fumaric acid relative to the total amount used, varied among the formulations. For HPO, the efficiency decreased with the addition of surfactants, with the control without surfactant showing the highest efficiency at 48.55%, and the 20% GML formulation showing the lowest at 10.60%. For HRO, an increase in encapsulation efficiency was observed with the addition of 15% PGM (57.11%) compared to the control (67.87%), while a decrease was noted with the 20% GML addition (31.18%). COM showed the highest efficiency in the control (84.30%), with a decrease observed upon surfactant addition, particularly with 20% GML (25.25%).

The effective loading capacity, indicating the actual amount of fumaric acid encapsulated, also demonstrated variation. For HPO, this capacity decreased with surfactant addition, with the lowest value observed at the 20% GML concentration. Similar trends were noted for HRO and COM, with the highest loading capacities associated with the control formulations without surfactants.

The approximate constancy of fumaric acid content, despite significant changes in encapsulation efficiency and loading capacity, can be attributed to the process parameters being optimized for a consistent drug payload. The spray-congealing process parameters, such as feed rate, atomization conditions, and cooling rate, are critical in determining the final particle characteristics [36]. These parameters likely compensate for variations in surfactant concentration, ensuring that the total fumaric acid content remains relatively stable across formulations.

The changes in encapsulation efficiency and loading capacity with surfactant addition are likely due to the surfactants’ influence on the process of particle formation and the physical properties of the lipid matrix. Surfactants can alter interfacial properties, which may affect the encapsulation process by changing the way fumaric acid is incorporated into the lipid matrix or by modifying the matrix’s ability to encapsulate the acid [37]. Furthermore, surfactants may impact the solidification rate of the molten lipid, which can directly affect the encapsulation efficiency and loading capacity [38].

While the fumaric acid content remains relatively constant due to optimized spray-congealing process parameters, the encapsulation efficiency and loading capacity are influenced by the surfactants’ effects on particle formation and matrix properties. Understanding these relationships is crucial for tailoring the process to achieve the desired outcomes in microparticle production.

#### 3.2.2. Particle Size

Understanding particle dimensions and shape is crucial for enhancing the performance of particulate systems, which relies on encapsulation efficiency, active ingredient release, and maintaining the bioactivity of substances [1]. Particle size is influenced by process elements such as cooling air temperature and velocity, atomization pressure, feed mixture flow rate through the atomizer, and atomizer nozzle specifications. Additionally, the carrier matrix’s lipid composition, affecting viscosity, the type and amount of surfactant in the mixture, the active ingredient’s properties, the bio-active to matrix ratio, and other factors are also crucial [39,40].

A detailed analysis of the experimental dataset reveals intricate relationships between the concentration of surfactants PGM and GML and the particle size distribution of spray-congealed microparticles formulated with different lipid carriers—HPO, HRO, and COM. The data compiled in Table 2 showed that within the same lipid, the addition of GML and PGM generally led to a reduction in the smallest (D_10_) and median (D_50_) particle sizes. This trend suggested that these surfactants were effective in creating smaller particles. For instance, in HPO, the D_10_ value decreased from 33.48 μm in the control to 19.22 μm with 20% GML, and the D_50_ value dropped from 81.38 μm to 66.68 μm, indicating a significant reduction in particle size.

However, the effect on the largest particle size (D_90_) was more complex and varied depending on the lipid used. In HPO, the D_90_ increased with GML addition, suggesting that while smaller particles were formed, larger ones also became more prevalent. Conversely, in COM, the D_90_ decreased with GML addition, indicating an overall reduction in particle size. The span, which reflects the width of the particle size distribution, also changed with surfactant addition. In HPO, the span increased from 1.40 in the control to 2.20 with 20% GML, indicating a broader distribution.

Comparing the effects of GML and PGM across different lipid carriers revealed that both surfactants tended to decrease D_10_ and D_50_ across all lipids. For example, in HRO, the D_10_ value decreased from 19.16 μm in the control to 13.58 μm with 20% GML, and the D_50_ value slightly increased from 62.40 μm to 66.82 μm. This suggests that GML could influence particle size reduction in a lipid-dependent manner. The span values also varied, with the broadest distribution observed in HPO and the narrowest in COM, indicating that both the surfactant concentration and lipid type played roles in determining the distribution width.

The span analysis, representing a measurement of the width of the size distribution, indicated a general trend towards more uniform particle sizes with the addition of surfactants, as shown by decreasing span values. However, the degree of this decrease varied, with the broadest distribution observed in HPO and the narrowest in COM, indicating that both surfactant concentration and lipid type influenced the distribution width.

In brief, the addition of GML and PGM surfactants to the lipid carriers during the encapsulation process significantly affected particle size distribution. Both surfactants reduced the smallest and median particle sizes, but their impact on the largest particle size and distribution width was dependent on the specific lipid used.

#### 3.2.3. Hot Stage Microscopy

**Figure 3:**
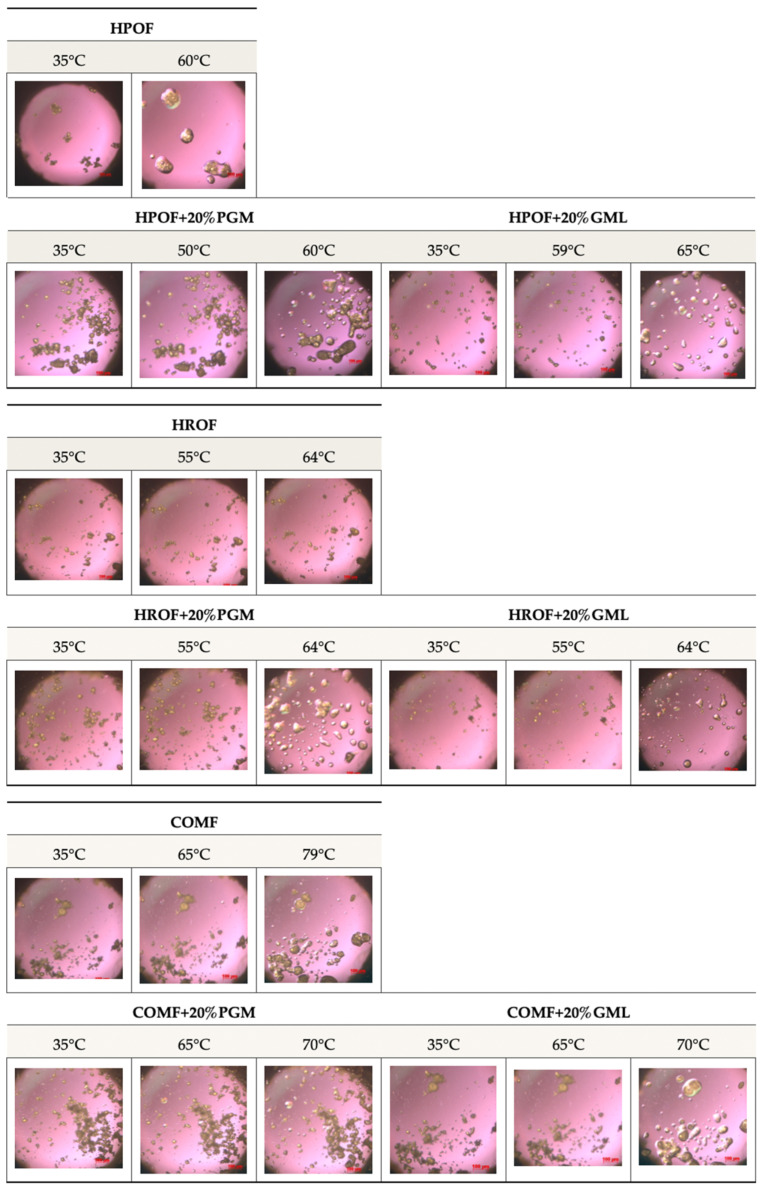
Selected hot-stage microscopy images captured at different phases of melting of 50%, *w*/*w* fumaric acid-loaded HPO, HRO, and COM microparticles with surfactants.

The meticulous selection of temperatures for the hot-stage microscopy analysis was instrumental in evaluating the release behaviour of the encapsulated fumaric acid, as shown in Figure 3. The temperatures were arbitrarily chosen to be below the melting points of the lipid carriers—HPO at 55 °C, HRO at 60 °C, and Compritol at 70 °C—to observe the initial state of the microparticles. The examination continued precisely at the melting points to capture the initial dissolution of the particulates, providing insights into the onset of the release process.

Furthermore, the analysis extended to temperatures above the melting points to study the complete melting behaviour of the microparticles. This comprehensive temperature profile enabled the observation of the transition from a solid state to a homogeneous melt, which is pivotal for understanding the release characteristics of the encapsulated fumaric acid. The rapid dissolution of the particulates at around 53 °C, as observed in the microscopy images, signifies the efficient release of the encapsulated substance.

The study of the release behaviour at temperatures below, at, and above the melting points of the lipid carriers is essential for assessing the performance of the microparticles in various conditions. It provides valuable data on the thermal stability and release kinetics of the encapsulated fumaric acid, which are critical parameters for its application in controlled-release formulations.

Moreover, the addition of 20% PGM and GML did not negatively affect the melting behaviour, indicating that these surfactants can be used to modify the properties of the microparticles without compromising the release characteristics of the encapsulated fumaric acid. This finding is significant for the development of microparticle formulations with tailored release profiles.

### 3.3. Determination of Fumaric Acid-Matrix Miscibility

The significant temperature fluctuations experienced during the spray congealing process may induce polymorphic transformations in both the encapsulated acid and the lipid matrix. Such transitions pose a substantial risk in food product formulation, potentially impacting the product’s stability and the profile of drug release. This part of the study investigates the influence of spray congealing and surfactants on the physical-chemical characteristics of the encapsulated fumaric acid and carrier, the spray-congealed microparticles, and the possible interaction between them.

#### 3.3.1. Thermal Analysis of Solid State of Fumaric Acid Loaded Solid Lipid Microparticles

**Figure 4:**
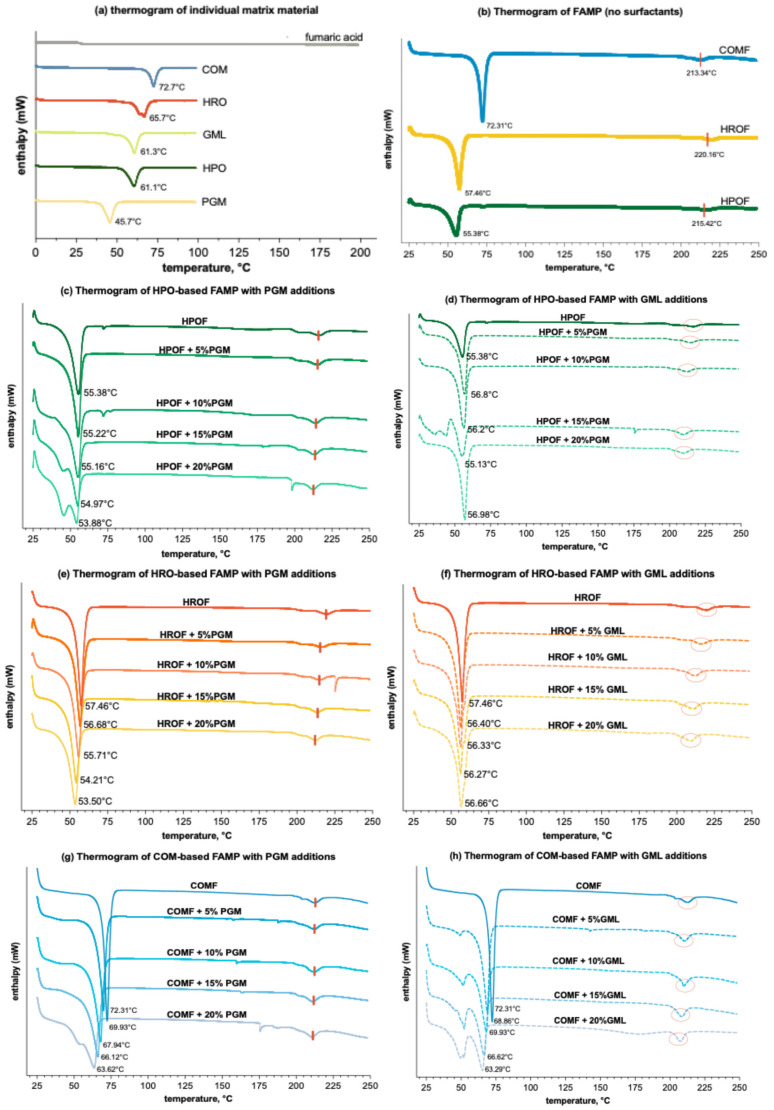
DSC thermograms (*n =* 3) of (**a**) individual materials; (**b**) spray-congealed microparticles with only lipid (HPO, HRO, or COM) and fumaric acid; (**c**) spray-congealed microparticles formulated with HPO or/and additions of PGM (incremental by 5%, *w*/*w*) and 50% fumaric acid; (**d**) spray-congealed microparticles formulated with HPO or/and additions of GML (incremental by 5%, *w*/*w*) and 50% fumaric acid; (**e**) spray-congealed microparticles formulated with HRO or/and additions of PGM (incremental by 5%, *w*/*w*) and 50% fumaric acid; (**f**) spray-congealed microparticles formulated with HRO or/and additions of GML (incremental by 5%, *w*/*w*) and 50% fumaric acid; (**g**) spray-congealed microparticles formulated with COM or/and additions of GML (incremental by 5%, *w*/*w*) and 50% fumaric acid; (**h**) spray-congealed microparticles formulated with COM or/and additions of GML (incremental by 5%, *w*/*w*) and 50% fumaric acid. For ease of differentiation, the three lipid and derivative formulations are color-coded: HPO-based formulations are represented by green lines, HRO-based formulations by orange/red lines, and COM-based formulations by blue lines. GML formulations were further distinguished with dashed lines to contrast with the solid lines used for PGM formulations. The endothermic peak of fumaric acid was marked by the vertical redline in (**b**,**c**,**e**,**g**) and circled in red in (**d**,**f**,**h**).

The objective of conducting the DSC test was to ascertain the polymorphic state of fumaric acid within the molten formulation. The DSC thermograms for the individual components and the range of formulations are depicted in Figure 4 below. The melting endotherms of COM, HRO, GML, HPO, and PGM were observed at 72.7 °C, 65.7 °C, 61.3 °C, 61.1 °C and 45.7 °C, respectively (Figure 4a). Only HRO exhibited two endotherms. The melting endotherm of fumaric acid revealed a meltdown peak at around 280 °C, corresponding to the melting point of fumaric acid at 289 °C.

At a consistent level of FA spray-congealed for 50%, it was observed that spray-congealing effectively suppressed the melting point of the lipid-based microparticles, though to a lesser extent for COM (Figure 4b). While the melting endothermic peak for HPO and HRO was each 61.6 °C and 65.7 °C, their corresponding microparticles, when produced through spray-congealing, fall to an endothermic peak at 55.38 °C and 56.46 °C, respectively. Interestingly, the endotherm of FAMP produced from COM peaked at 72.31 °C, which did not deviate from the melting endotherm of the lipid COM itself at 72.7 °C. For all the formulations with/without surfactants, the acid retains itself as insoluble particles in the microparticles, supported by the presence of the fumaric acid peak in all formulations (Figure 4b–h), though the endothermic peak of fumaric acid was not very obvious in the graphics (marked with a shortened red vertical line in Figure 4b,c,e,g, and circled in red in Figure 4d,f,h). At a high concentration of a 50% (*w*/*w* lipid and surfactant) acid load, the solubility threshold for fumaric acid was surpassed, preventing complete dissolution in the lipid matrix. This is proof that it remained as a crystalline dispersion within the liquid matrix.

This phenomenon of matrix melting point reduction by the presence of solutes is known as the colligative property [41]. The extent of this reduction, however, is directly proportional to the solute’s concentration. As the concentration of fumaric acid was uniformed in the formulation, it can be inferred that the magnitude of melting endotherms at 210 °C signifies the extent of its solubility in the matrix. In this study, nearly all HRO and HPO formulation peaks showed a marked reduction in melting point (*p* < 0.05), indicating a significant effect. However, the melting point depression observed in the COM formulations was not statistically significant (*p* ≥ 0.05) (Appendix A, Table A1). This suggests that fumaric acid has higher solubility in both HPO and HRO, given the same quantity. The reason might be attributed to the unique triglyceride makeup of palm stearin lipid and hydrogenated rapeseed oil, which foster more disordered and crystalline structures. This structure helps in avoiding the expulsion of the encapsulated bioactive compounds during the solidification phase [42].

In addition, the presence of surfactants has a consistent effect of lowering the melting points of the three lipids, albeit to varying degrees. Specifically, PGM uniformly reduces the melting points across all lipids. In contrast, GML exhibits a more complex behaviour: it slightly raises the melting point when included in the formulations of HROF and HPOF, yet it significantly decreases the melting point in the case of COM formulations. The impact of surfactants on melting points seems to follow a linear trend: an increase in the proportion of additives in a formulation led to continual reduction in the melting point.

#### 3.3.2. Powder X-ray Diffraction Studies

In the spray-congealing process, a drug like fumaric acid may dissolve either fully or partially within the molten matrix, leading to various states in the final product: molecular, amorphous, or crystalline. The state of fumaric acid can significantly impact its solubility, stability, and release characteristics. It recognises that a drug’s crystallinity—whether partially crystalline, amorphous, or fully crystalline—affects its water solubility and thermodynamic stability. Specifically, partially crystalline or amorphous forms are more water soluble and less stable compared to their crystalline counterparts, which is a critical consideration in the context of drug release and product shelf life [43].

**Figure 5:**
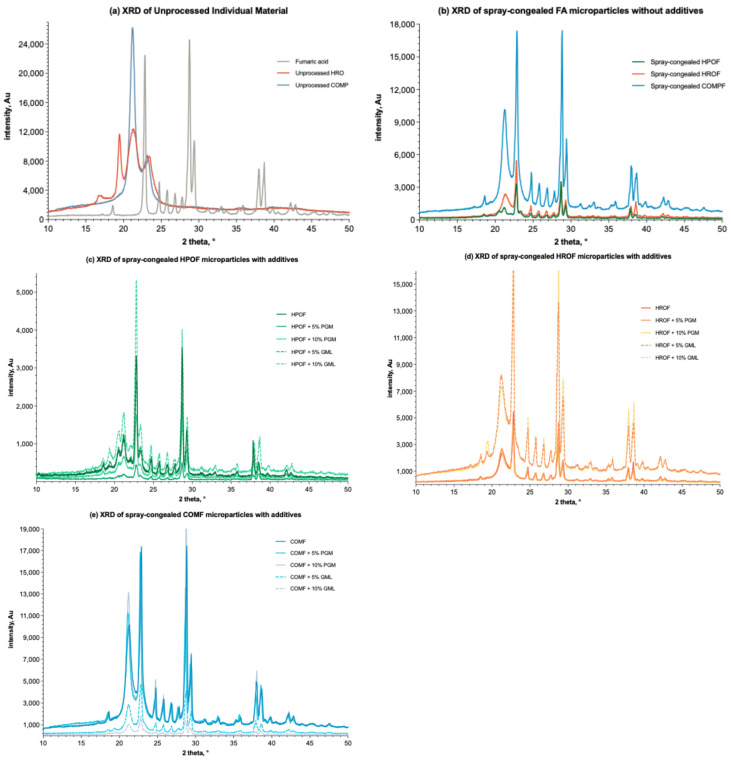
XRD spectra (*n* = 3) of (**a**) unprocessed individual materials; (**b**) spray-congealed microparticles with only lipid (HPO, HRO, or COM) and fumaric acid; (**c**) spray-congealed microparticles formulated with HPO or/and additions of PGM and GML (incremental by 5%, *w*/*w*, as labelled) and 50% fumaric acid; (**d**) spray-congealed microparticles formulated with HRO or/and additions of PGM and GML (incremental by 5%, *w*/*w*, as labelled) and 50% fumaric acid; (**e**) pray-congealed microparticles formulated with COM or/and additions of PGM and GML (incremental by 5%, *w*/*w*, as labelled) and 50% fumaric acid.

**Figure 6:**
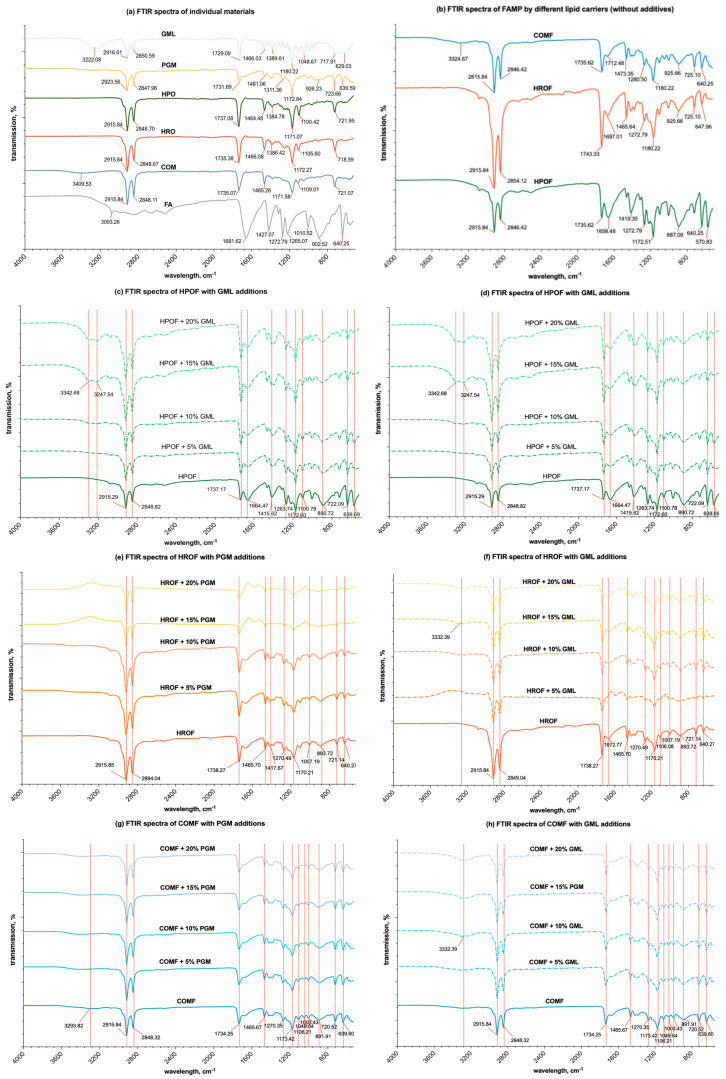
FTIR spectra of (**a**) individual materials; (**b**) spray-congealed microparticles with only lipid (HPO, HRO, or COM) and fumaric acid; (**c**) FAMP formulated with HPO or/and additions of PGM; (**d**) FAMP formulated with HPO or/and additions of GML; (**e**) FAMP formulated with HRO or/and additions of PGM; (**f**) FAMP formulated with HRO or/and additions of GML; (**g**) FAMP formulated with COM or/and additions of PGM; (**h**) FAMP formulated with COM or/and additions of GML.

The diffraction patterns of individual components and various formulations are shown in Figure 5. The characteristic peaks of fumaric acid, corresponding to its crystallographic planes (PDF#15-1187), are sharp and distinct at 2-Theta values of 21.1°, 24.4°, 28.9°, 38.2°, and 38.7° (Figure 6a) [44]. These peaks are evident in all formulation spectra, confirming the presence of crystalline fumaric acid and corroborating the DSC results, which indicate that fumaric acid is dispersed within the formulations. Among the lipid-only formulations, microparticles encapsulated with COM displayed the highest peak intensities, suggesting a higher degree of crystallinity (Figure 5b). This is particularly pronounced at a high drug load of 50% *w*/*w*, where the peak intensity of the COM formulation significantly differs from those of HPO and HRO formulations. The disparity in crystallinity is believed to stem from the conditions available for fumaric acid molecules to rearrange into a crystal lattice during the cooling cycle. This hypothesis is supported by the DSC results (Figure 4), which show a more pronounced endothermic peak for fumaric acid in the COM matrix compared to HPO and HRO, indicating a more stable crystalline form.

The chemical structure of COM, a glyceride of saturated fatty acids, likely facilitates the formation of a stable crystal lattice for fumaric acid due to its rigid and orderly matrix. This is supported by the work of Teeranachaideekul et al. [8] and Keck et al., 2021 [45], where researchers discussed the influence of lipid matrix composition on drug crystallinity. In contrast, the lower peak intensities observed for HPO and HRO suggest a less crystalline environment, potentially due to differences in fatty acid composition and saturation levels. The rigid matrix of HPO might restrict the mobility of fumaric acid molecules, impacting lattice formation, while the distinct fatty acid composition of HRO may create a less favourable environment for crystallisation. This is in line with the findings of Hancock and Zografi [46], who detailed how the fatty acid chain composition can impact crystallization. The higher crystallinity in the COM matrix is expected to affect the fumaric acid release and stability of the microparticles. A more crystalline substance typically has lower solubility and slower dissolution rates, potentially leading to a slower drug release profile and enhanced stability. Thus, the choice of COM as a lipid carrier in the spray-congealing process influences not only the crystallinity of the entrapped fumaric acid but also the performance characteristics of the final product.

The addition of PGM and GML to the formulations (Figure 5c–e) introduced a variable that affects the crystallinity of fumaric acid. As the concentration of PGM and GML increases in increments of 5% *w*/*w*, the XRD spectra reveal changes in peak intensities. For instance, the addition of 5% GML to COMF results in a noticeable increase in peak intensity compared to the unprocessed COMF, indicating a potential increase in crystallinity. This trend is observed across the lipid carriers, indicating that the surfactants may enhance the crystallization of fumaric acid within the matrix, as suggested by the studies of Muller and Keck [47] on the effects of surfactants on drug crystallization.

#### 3.3.3. Flourier-Infrared Spectroscopy

The FTIR spectroscopy analysis of various substances, including unprocessed fumaric acid, lipid matrix materials (HPO, HRO, and COM), and surfactants (GML, PGM), as well as spray-congealed fumaric acid-loaded solid-lipid microparticles with and without additives, was conducted on solid-state samples. This study anticipates that the interactions observed will be similar in the molten state, potentially impacting viscosity.

As shown in Figure 6a, fumaric acid’s spectrum was marked by prominent carbonyl peaks (1650–1750 cm^−1^) and C-H stretches (2800–3100 cm^−1^). COM’s spectrum was distinguished by a unique wide O-H stretch absorption at 3420.85 cm^−1^, not observed in HRO and HPO. The C=O stretch absorption peaks for HPO, HRO, and COM were identified at 1737.08 cm^−1^, 1735.38 cm^−1^, and 1735.07 cm^−1^, respectively, indicating slight variations in the molecular structure of these lipid matrix materials, as referenced by Wallace [48].

The FTIR spectral analysis (Figure 6b–g) of spray-congealed solid-lipid microparticles containing fumaric acid and varying concentrations of the additives PGM and GML showed that the spectra of blends in various proportions were largely similar to the respective combinations of the spectra of the individual components. There were only observable differences with the increasing concentrations of GML in the formulation. The absence of noticeable spectra differences with the presence of PGM in the formulation, however, does not exclude the possibility of interactions between the materials when blended in the molten state,

For the HPO lipid carrier, as shown in Figure 6c,d, the C = O absorption band exhibited a gradual shift from 1737.08 cm^−1^ to 1735.20 cm^−1^, with increasing PGM concentration up to 20% *w*/*w*. A more pronounced shift to 1732.54 cm^−1^ was observed with increasing GML concentration. Additionally, the introduction of GML led to the appearance of O-H stretch absorptions at 3324.68 cm^−1^ and 3255.25 cm^−1^ into two smaller peaks, suggesting a modest level of hydrogen bonding with fumaric acid. In the case of the HRO lipid carrier (Figure 6e,f), similar trends were observed with the C = O absorption band shifting from 1735.38 cm^−1^ to 1734.67 cm^−1^ with PGM and to 1732.62 cm^−1^ with GML. The addition of GML also resulted in broad O-H absorption at 3262.97 cm^−1^, indicating hydrogen bonding, albeit to a lesser extent than with HPO. For the COM lipid carrier (Figure 6g,h), the C = O absorption band shifted from 1735.07 cm^−1^ to 1734.32 cm^−1^ with PGM and to 1730.67 cm^−1^ with GML. The addition of GML induced a broad O-H absorption at 3316.6 cm^−1^, suggesting the presence of hydrogen bonding, but again, to a lesser degree than observed with HPO.

These findings indicate that while hydrogen bonding occurs between the lipid carriers and fumaric acid, the degree of bonding varies with the type of lipid carrier and the additive used. The addition of GML to the COM lipid carrier showed the least degree of hydrogen bonding compared to its effect on HPO and HRO, which could have implications for the formulation and performance of the SLMs. The wide O-H stretch band characteristic of COM in spray-congealed microparticles, which also include a polymeric additive, exhibited only slight variations. It comes as no surprise that the interaction through hydrogen bonding between COM and the additives is quite restricted. This is due to the fact that COM is a substantially large diglyceride molecule, featuring a single hydroxyl group that is embedded within two lengthy and substantial behenate chains. Such a molecular structure leads to significant steric hindrance, which in turn impedes the establishment of robust hydrogen bonds with the carbonyl groups of the additives.

### 3.4. FAMP Release Studies

The in vitro dissolution profile of acid release was investigated using distilled water as the dispersion medium to assess the release rates of different formulations. As previously mentioned, carriers can be utilised to manipulate drug release profiles for various applications. Passerini et al. [40] successfully used Compritol 888 ATO as a carrier to control the release of a highly water-soluble drug, theophylline, in the form of microparticles.

To simulate the baking situation, the release studies were divided into two phases. In the first phase, all 27 spray-congealed microparticle formulations underwent a temperature-dependent release test. Following the results of the temperature-dependent release study and the encapsulation efficiency assessments of the microparticles, a selection of samples was chosen for the subsequent time-dependent dissolution test.

Briefly, the microparticles were subjected to water bath at 35 °C, 53 °C, 67 °C, and 90 °C for 15 min. Each temperature profile was arbitrarily chosen to model the baking scenario of dough: 35 °C was chosen as the common maximum dough proofing temperature in the industry; 53 °C was when the temperature just below the lowest melting point of our samples; 67 °C was above the melting points for both lipid HPO and HRO; 90 °C was close to the maximum temperature reached for the internal temperature of the baking dough, and it was set to ensure all the acid has been effectively released at the end of the release modelling.

#### 3.4.1. Temperature-Dependent Release Profile

**Figure 7:**
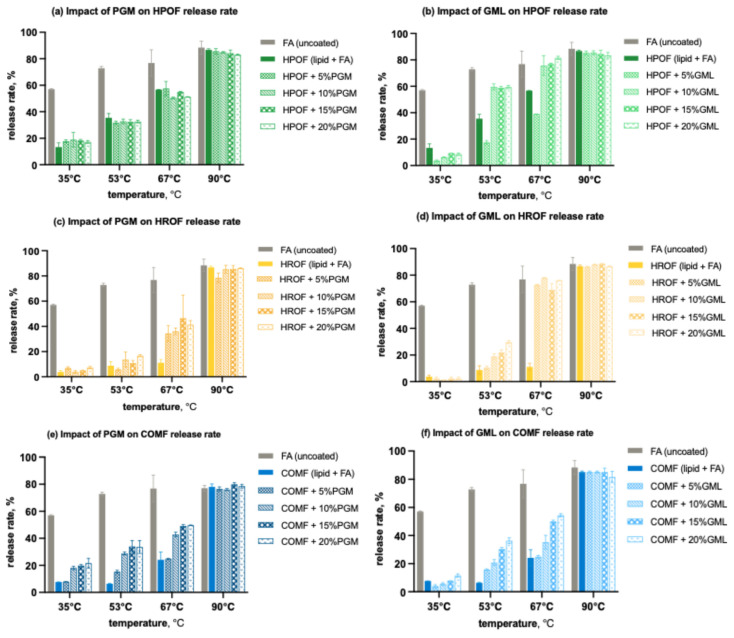
Effect of surfactants on the temperature-dependent release rate of spray-congealed FA microparticles for 15 min: (**a**) effect of PGM on the release rate of HPO-based FAMP; (**b**) effect of GML on the release rate of HPO-based FAMP; (**c**) effect of PGM on the release rate of HRO-based FAMP; (**d**) effect of GML on the release rate of HRO-based FAMP; (**e**) effect of PGM on the release rate of COMP-based FAMP; (**f**) effect of GML on the release rate of COMP-based FAMP.

The release profiles for all formulations, as illustrated in Figure 7 demonstrated significant variations. Pure fumaric acid, with its modest solubility in water at lower temperatures, showed a release of just under 60% at 35 °C. This percentage increased to approximately 72% at 53 °C, 79% at 67 °C, and peaked at 90% at 90 °C. These results were somewhat lower than anticipated based on the solubility profile of fumaric acid, likely due to the constraints of the experimental setup.

Microencapsulation proved universally effective in regulating the release of fumaric acid in water, outperforming the release profile of uncoated fumaric acid across all three lipid carriers, regardless of their encapsulation efficiencies. With HPO as the carrier, GML was notably more successful in slowing the release of acid at 35 °C, contrasting with the higher release levels observed with PGM at all concentrations (Figure 7a,b). This outcome is intriguing, given that microparticles containing GML had a lower encapsulation efficiency, potentially attributed to GML’s relatively more hydrophobic nature. Above 53 °C, formulations with GML exhibited a higher release profile compared to those with PGM. By 90 °C, the majority of the fumaric acid had been released.

For HRO, GML similarly outperformed in impeding acid release at 35 °C, with all four concentrations showing lower release profiles than the surfactant-free formulation (Figure 7c,d). However, beyond 53 °C, the effectiveness of both GML and PGM in controlling release diminished, with GML showing a less pronounced effect than PGM. At 90 °C, most of the fumaric acid was released.

In the case of COM, GML was again more effective in impeding acid release (Figure 7e,f). As temperatures rose, the effectiveness of both PGM and GML in suppressing release decreased across all concentrations, with a higher release rate compared to the additive-free formulation. The distinction between PGM and GML was less pronounced for COM than for HPO and HRO, possibly due to COM’s inherently higher encapsulation efficiency. By the time the temperature reached 90 °C, most of the fumaric acid had been released.

#### 3.4.2. Time-Dependent Release Profile

Based on the release profile and encapsulation efficiency, only formulations with lipids HRO and COM, containing 5% and 10% PGM and GML, were selected for subsequent experiments. It was observed that beyond a 10% concentration, both encapsulation efficiency and temperature-dependent release profiles deteriorated rapidly compared to the spray-congealed microparticle formulations without surfactants. Unencapsulated raw fumaric acid was also tested for comparative purposes.

**Figure 8:**
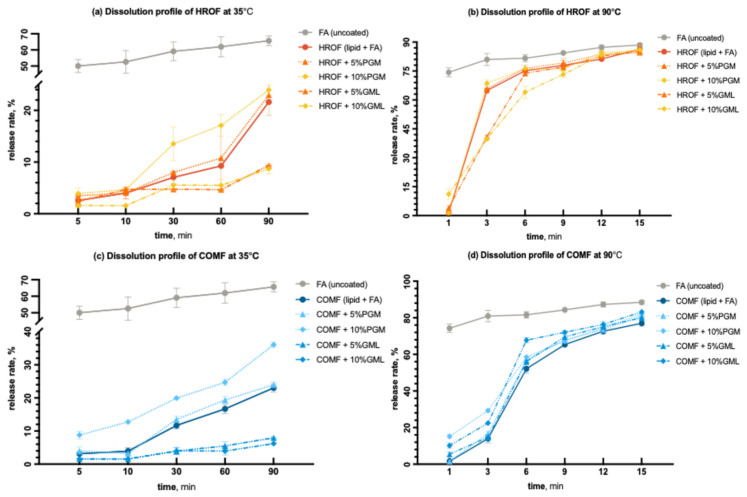
Dissolution profiles of (**a**) HROF with variations in PGM and GML at 35 °C; (**b**) HROF with variations in PGM and GML at 90 °C; (**c**) COMF with variations in PGM and GML at 35 °C; (**d**) COMF with variations in PGM and GML at 90 °C.

At 35 °C, 50% of uncoated fumaric acid was released within 5 min, and 60% was released within 90 min (Figure 8a,c). This aligns with the known dissolution profile of fumaric acid, which is only slightly soluble in an aqueous environment at lower temperatures [49]. For both HRO and COM lipids, GML slowed the release rates compared to formulations without additives, despite contributing to lower encapsulation efficiency (Table 1). This effect was attributed to GML’s more hydrophobic nature compared to PGM, which may offset the lower encapsulation efficiency. At 90 °C, for HRO, PGM was more effective in impeding the release of fumaric acid within the first 6 min compared to the HRO formulation without any additive (Figure 8b,d). Beyond 9 min, the trends converged, and most of the acid was released by the end of the 15 min period.

For COM at 90 °C, neither PGM nor GML was more effective in impeding the release of fumaric acid compared to the COM formulation without any additive (Figure 8c,d). The release profiles of all COM formulations were similar as a function of time, although the formulation without any additive released the acid more rapidly. This could be due to its higher encapsulation efficiency. At higher temperatures, the impact of encapsulation efficiency on the release profile was more pronounced.

## 4. Conclusions

The current study offers a detailed examination of how lipid carriers and surfactants influence the spray-congealing process for the encapsulation of fumaric acid. The results objectively highlight that the choice of lipid materials and the incorporation of surfactants notably affect the rheological behaviour, encapsulation efficiency, particle size distribution, and release profiles of the produced solid lipid microparticles.

The findings indicate that the viscosity and stability of the molten mixtures are significantly influenced by the presence of surfactants. This influence subsequently impacts the microencapsulation process and the subsequent release characteristics of fumaric acid. The observed temperature-dependent and time-dependent release profiles suggest the potential for tailoring the release kinetics to meet the specific demands of applications such as the baking industry, where controlled release at precise stages is essential.

The empirical data consistently show that the lipid carriers—hydrogenated palm stearin (HPO), hydrogenated rapeseed oil (HRO), and Compritol (COM)—as well as the surfactants propylene glycol monostearate (PGM) and glyceryl monolaurate (GML) influence the crystallinity of fumaric acid. This influence may result in a slower release profile and enhanced stability of the encapsulated fumaric acid—a factor that is crucial for its performance in food products.

Furthermore, this study presents an objective analysis of the particle size distribution and thermal behaviour of the microparticles, which are pivotal for assessing their efficacy in controlled-release applications. The results confirm that the spray-congealing process is effective for encapsulating fumaric acid and can modulate its release in accordance with the requirements of the baking process.

In conclusion, the spray-congealing technique emerges as a feasible method for encapsulating fumaric acid in SLMs. The process parameters, along with the properties of the lipid carriers and the effects of surfactants, play a critical role in shaping the characteristics of the microparticles and their applicability in specific uses. Future studies should concentrate on fine-tuning these parameters to optimize the functionality of fumaric acid in food products, ensuring that product quality is preserved and enhanced.

## Figures and Tables

**Table 1:** Acid content, loading capacity, and encapsulation efficiency of ternary spray-congealed formulations. (average ± SD; *n* = 3).

Formulation		Acid Content %	Encapsulation Efficiency, %	Effective Loading Capacity, %
Lipid	Surfactant			
HPO	— (control) only FA	52.40 ± 0.90 ^a^	48.55 ± 3.09 ^a^	25.41 ± 1.68 ^a^
	5%PGM	51.82 ± 1.77 ^a^	37.42 ± 6.06 ^ab^	19.50 ± 5.38 ^ab^
	10%PGM	52.62 ± 0.08 ^a^	37.13 ± 1.12 ^ab^	19.54 ± 0.79 ^ab^
	15%PGM	52.27 ± 0.53 ^a^	28.97 ± 0.82 ^bcd^	15.15 ± 0.82 ^bcd^
	20%PGM	50.70 ± 0.21 ^a^	24.28 ± 0.67 ^be^	12.31 ± 0.41 ^be^
	5%GML	51.39 ± 0.38 ^a^	32.82 ± 4.07 ^abc^	16.85 ± 2.78 ^abc^
	10%GML	51.10 ± 0.64 ^a^	18.95 ± 3.66 ^ce^	9.71 ± 2.82 ^ce^
	15%GML	50.86 ± 0.54 ^a^	13.05 ± 0.33 ^de^	6.64 ± 0.34 ^de^
	20%GML	51.12 ± 0.85 ^a^	10.60 ± 0.83 ^e^	5.43 ± 0.73 ^e^
HRO	— (control) only FA	52.38 ± 0.14 ^a^	67.87 ± 0.49 ^a^	35.55 ± 0.50 ^a^
	5%PGM	51.49 ± 0.50 ^ac^	63.31 ± 0.88 ^ab^	32.59 ± 0.20 ^ab^
	10%PGM	50.14 ± 0.38 ^c^	58.35 ± 1.85 ^b^	29.25 ± 1.00 ^bc^
	15%PGM	50.18 ± 0.27 ^c^	57.11 ± 0.54 ^b^	28.66 ± 0.17 ^c^
	20%PGM	50.47 ± 0.05 ^c^	57.30 ± 0.73 ^b^	28.92 ± 0.56 ^c^
	5%GML	50.18 ± 0.21 ^c^	69.87 ± 2.26 ^a^	35.07 ± 1.81 ^a^
	10%GML	50.74 ± 0.10 ^bc^	48.40 ± 1.81 ^c^	24.56 ± 1.37 ^d^
	15%GML	52.11 ± 0.27 ^ab^	39.60 ± 0.11 ^d^	20.63 ± 0.23 ^e^
	20%GML	52.12 ± 0.05 ^ab^	31.18 ± 0.59 ^e^	16.25 ± 0.41 ^f^
COM	— (control) only FA	52.27 ± 0.28 ^a^	84.30 ± 2.81 ^a^	44.07 ±2.41 ^a^
	5%PGM	50.10 ± 0.01 ^b^	79.05 ± 0.17 ^a^	39.61 ± 0.13 ^b^
	10%PGM	50.36 ± 0.02 ^b^	69.11 ± 1.02 ^b^	34.80 ± 0.75 ^c^
	15%PGM	50.60 ± 0.26 ^b^	57.07 ± 0.92 ^c^	28.88 ± 0.86 ^d^
	20%PGM	50.86 ± 0.21 ^b^	43.61 ± 0.28 ^d^	22.18 ± 0.33 ^e^
	5%GML	50.78 ± 0.48 ^b^	82.93 ± 1.34 ^a^	42.10 ± 0.40 ^ab^
	10%GML	50.32 ± 0.18 ^b^	69.77 ± 1.57 ^b^	35.11 ± 1.29 ^c^
	15%GML	50.75 ± 0.18 ^b^	35.94 ± 0.77 ^e^	18.24 ± 0.46 ^e^
	20%GML	50.44 ± 0.11 ^b^	25.25 ± 0.63 ^f^	12.74 ± 0.48 ^f^

Significant differences were analysed within samples of the same lipid carrier; mean values with different superscript letters were significantly different.

**Table 2:** Influence of additives on particle size distribution of microparticles (average ± SD; *n* = 5).

Formulation		Particle Size Distribution (*n* = 5)			
Lipid	Surfactant	D_10_, μm	D_50_, μm	D_90_, μm	Span
HPO	— (control) only acid	33.48 ± 1.60 ^a^	81.38 ± 2.41 ^b^	147.4 ± 4.56 ^e^	1.40 ± 0.01 ^e^
	5%PGM	25.92 ± 1.52 ^bc^	74.32 ± 4.67 ^c^	170.5 ± 3.54 ^d^	2.43 ± 0.39 ^ac^
	10%PGM	23.96 ± 0.88 ^bd^	70.32 ± 1.46 ^cd^	159.6 ± 2.19 ^de^	1.94 ± 0.07 ^d^
	15%PGM	22.96 ± 0.62 ^cde^	66.70 ± 1.72 ^d^	156.4 ± 4.22 ^de^	1.99 ± 0.05 ^d^
	20%PGM	21.50 ± 0.94 ^df^	64.88 ± 1.71 ^d^	161.8 ± 7.19 ^d^	2.16 ± 0.05 ^cd^
	5%GML	26.38 ± 2.39 ^b^	88.04 ± 3.95 ^a^	244.6 ± 13.39 b	2.48 ± 0.08 a
	10%GML	20.90 ± 2.11 ^ef^	87.82 ± 2.35 ^a^	259.2 ± 7.33 ^a^	2.72 ± 0.10 ^a^
	15%GML	20.40 ± 1.26 ^ef^	75.42 ± 2.61 ^c^	195.6 ± 4.45 ^c^	2.32 ± 0.07 ^bc^
	20%GML	19.22 ± 0.46 ^f^	66.68 ± 0.65 ^d^	165.8 ± 3.70 ^d^	2.20 ± 0.07 ^cd^
HRO	— (control) only FA	19.16 ± 0.58 ^b^	62.40 ± 1.49 ^fg^	145.00 ± 8.72 ^ce^	2.02 ± 0.13 ^bd^
	5%PGM	19.02 ± 0.52 ^b^	73.50 ± 0.91 ^b^	187.00 ± 10.39 ^b^	2.32 ± 0.07 ^bc^
	10%PGM	18.72 ± 0.84 ^b^	69.60 ± 2.56 ^c^	155.20 ± 21.59 ^cd^	2.20 ± 0.07 ^cd^
	15%PGM	17.06 ± 0.68 ^c^	65.32 ± 1.59 ^de^	140.00 ± 4.18 ^de^	1.88 ± 0.04 ^d^
	20%PGM	16.64 ± 0.35 ^c^	66.12 ± 1.05 ^d^	160.60 ± 5.68 ^d^	2.48 ± 0.08 ^a^
	5%GML	19.06 ± 0.26 ^b^	60.14 ± 0.29 ^g^	134.80 ± 3.83 ^e^	1.93 ± 0.08 ^d^
	10%GML	20.18 ± 0.36 ^a^	86.36 ± 1.29 ^a^	212.40 ± 5.13 ^a^	2.23 ± 0.06 ^ab^
	15%GML	17.10 ± 0.19 ^c^	63.12 ± 0.64 ^ef^	146.80 ± 3.96 ^ce^	2.06 ± 0.05 ^ad^
	20%GML	13.58 ± 0.16 ^d^	66.82 ± 0.33 ^d^	143.60 ± 2.5 ^ce^	1.94 ± 0.04 ^cd^
COM	— (control) only FA	23.20 ± 0.60 ^a^	85.38 ± 1.21 ^a^	203.40 ± 5.60 ^a^	2.11 ± 0.04 ^ab^
	5%PGM	18.08 ± 0.33 ^c^	60.98 ± 0.58 ^cde^	135.80 ± 1.30 ^bd^	1.93 ± 0.02 ^cd^
	10%PGM	15.96 ± 0.49 ^d^	61.86 ± 0.47 ^cd^	144.00 ± 3.24 ^b^	2.07 ± 0.07 ^abc^
	15%PGM	15.60 ± 0.38 ^d^	57.16 ± 0.95 ^ef^	136.80 ± 9.26 ^bc^	2.12 ± 0.16 ^a^
	20%PGM	14.04 ± 1.02 ^e^	44.48 ± 3.45 ^g^	92.30 ± 11.37 ^e^	1.75 ± 0.09 ^ef^
	5%GML	20.48 ± 0.73 ^b^	57.10 ± 1.64 ^f^	125.60 ± 4.16 ^cd^	1.84 ± 0.03 ^de^
	10%GML	18.42 ± 0.19 ^c^	60.16 ± 1.33 ^df^	136.60 ± 4.16 ^bc^	1.96 ± 0.04 ^bd^
	15%GML	17.71 ± 0.56 ^c^	67.37 ± 2.97 ^b^	127.90 ± 4.72 ^cd^	1.64 ± 0.01 ^f^
	20%GML	17.76 ± 0.21 ^c^	64.47 ± 1.55 ^bc^	123.30 ± 4.80 ^d^	1.64 ± 0.05 ^f^

Significant differences were analysed within samples of the same lipid carrier within the same column; mean values with different superscript letters were significantly different.

## Data Availability

The original contributions presented in the study are included in the article, further inquiries can be directed to the corresponding author.

## Appendix A

**Table A1:** Melting points and flow characteristics of potential selection of lipid-based materials.

Material	Main Component	Melting Pt, °C	Spraying Temperature, °C	Molten Feed Bath Temperature, °C
Hydrogenated Rapeseed Oil	Rapeseed	65.67 ± 1.82	70.67	75.67
Hydrogenated Palm Oil I (flakes)	Palm	61.26 ± 0.73	66.26	71.26
Compritol 888 ATO	glycerol behenate	72.72 ± 0.47	77.72	82.72
